# Higher Plant Calreticulins Have Acquired Specialized Functions in Arabidopsis

**DOI:** 10.1371/journal.pone.0011342

**Published:** 2010-06-28

**Authors:** Anna Christensen, Karin Svensson, Lisa Thelin, Wenjing Zhang, Nico Tintor, Daniel Prins, Norma Funke, Marek Michalak, Paul Schulze-Lefert, Yusuke Saijo, Marianne Sommarin, Susanne Widell, Staffan Persson

**Affiliations:** 1 Department of Biochemistry, Center for Chemistry and Chemical Engineering, Lund University, Lund, Sweden; 2 Department of Plant Physiology, Umeå Plant Science Center, Umeå University, Umeå, Sweden; 3 Department of Cell and Organism Biology, Lund University, Lund, Sweden; 4 Department of Plant Microbe Interactions, Max-Planck-Institute for Plant Breeding Research, Cologne, Germany; 5 Department of Biochemistry, University of Alberta, Edmonton, Canada; 6 Max-Planck-Institute for Molecular Plant Physiology, Wissenschaftspark Golm, Potsdam, Germany; Iowa State University, United States of America

## Abstract

**Background:**

Calreticulin (CRT) is a ubiquitous ER protein involved in multiple cellular processes in animals, such as protein folding and calcium homeostasis. Like in animals, plants have evolved divergent CRTs, but their physiological functions are less understood. Arabidopsis contains three CRT proteins, where the two CRTs AtCRT1a and CRT1b represent one subgroup, and AtCRT3 a divergent member.

**Methodology/Principal Findings:**

Through expression of single Arabidopsis family members in CRT-deficient mouse fibroblasts we show that both subgroups have retained basic CRT functions, including ER Ca^2+^-holding potential and putative chaperone capabilities. However, other more general cellular defects due to the absence of CRT in the fibroblasts, such as cell adhesion deficiencies, were not fully restored. Furthermore, *in planta* expression, protein localization and mutant analyses revealed that the three Arabidopsis CRTs have acquired specialized functions. The AtCRT1a and CRT1b family members appear to be components of a general ER chaperone network. In contrast, and as recently shown, AtCRT3 is associated with immune responses, and is essential for responsiveness to the bacterial Pathogen-Associated Molecular Pattern (PAMP) elf18, derived from elongation factor (EF)-Tu. Whereas constitutively expressed *AtCRT1a* fully complemented *Atcrt1b* mutants, *AtCRT3* did not.

**Conclusions/Significance:**

We conclude that the physiological functions of the two CRT subgroups in Arabidopsis have diverged, resulting in a role for AtCRT3 in PAMP associated responses, and possibly more general chaperone functions for AtCRT1a and CRT1b.

## Introduction

The endoplasmic reticulum (ER) localized protein calreticulin (CRT) is an important component for protein folding and Ca^2+^ homeostasis in the ER of animal cells (for review see [1]). In addition, animal CRTs have been implicated in more than 40 other cellular functions, demonstrating the versatility of this protein [2].

More specifically, CRT-deficient mouse fibroblasts are impaired in ER Ca^2+^-storage, and in bradykinin (BK)-induced Ca^2+^-releases from the ER [3]. These deficiencies are attributed to the two main functions of CRT, namely its Ca^2+^-buffering capacity in the ER, and its role in protein folding. The latter is presumably important for correct folding of the plasma membrane associated BK receptor [3]. Consequently, mutations in CRT result in lower levels of the BK receptor, and therefore impairment in BK-induced Ca^2+^ releases from the ER. In addition, the *crt^−/−^* fibroblasts also exhibit defects in cell adhesiveness [4], which is linked to the decrease in ER Ca^2+^-storage in these cells. The ER Ca^2+^ is utilized to maintain cell adhesiveness by regulation of adhesion-specific proteins, such as fibronectin and vinculin, and by modulation of tyrosine phosphorylation cascades [5]; [6].

As compared to the functions of CRT in animal cells, the role of plant CRTs is less clear [7]. Analogous to the animal protein, CRT may affect the ER Ca^2+^ homeostasis in tobacco cells *in vitro* [8], and *in vivo* [9]. In addition, over-production of a maize CRT in tobacco cell suspensions improved growth of cells grown in high Ca^2+^-medium [10]. Conversely, *Arabidopsis* plants over-expressing a maize CRT showed reduced leaf chlorosis when grown on Ca^2+^-depleted media compared to wild-type control plants [9]. These data suggest that CRT has an important role in buffering and modulating ER Ca^2+^ also in higher plants. Recently a plant *CRT*, *Arabidopsis thaliana* (*At)CRT1a*, expressed in CRT-deficient mouse fibroblast was able to restore the Ca^2+^-holding capacity to levels comparable to control fibroblast [11]. These results corroborate the results obtained *in planta*, and suggest a conservation of basic CRT functions between the two kingdoms.

CRT is also believed to harbor chaperone-like functions in plants (for review see [12]. The best evidence for such a function is that CRT may form stress-induced, i.e. heat-shock-induced, protein complexes in tobacco leaves, suggesting that it may bind to unfolded proteins and therefore possibly function as a molecular chaperone [13]. In addition, [11] showed that AtCRT1a restored putative folding deficiencies in CRT-deficient mouse fibroblasts.

The expression and localization of CRTs may vary depending on tissue and developmental stage. CRT appears to be present in most plant cells and tissues [12], but seems to be expressed abundantly in floral tissues [14], and in germinating seeds [13]. In *Arabidopsis* flowers, CRT is mainly expressed in secreting nectaries, endosperm ovules early in development, and in the posterior of pollen sacs [15]. CRT levels are also often elevated directly after fertilization and during early embryogenesis in a variety of plant species [13] to [18]. CRT appears to mainly reside in the ER [14]; [19], and in the Golgi [14]; [20]. Other studies have also localized CRT to the nuclear envelope in plant cells [13]; [21]. Interestingly, CRT was preferentially localized to cell periphery-associated parts of ER, such as plasmodesmata, in maize roots [22]. Similar localizations have also been reported in *Nicotiana benthamiana* [16].

Both plants and mammals appear to contain two subgroups of CRT proteins [15]; . For example, Arabidopsis holds three CRT family members that are classified into an AtCRT1a/1b, and an AtCRT3 group, on the basis of sequence homology [24]. However, little is known about possible differences among the members of the plant CRT family regarding their functions, expression, and subcellular localization [12]. One study demonstrated that AtCRT1a has retained basal CRT functions shared across different kingdoms as assessed by complementation of a CRT-deficient mouse fibroblast system [11]. *Atcrt1a* mutant plants show lower tolerance to tunicamycin, an inhibitor of N-linked glycosylation that causes ER stress, referred to as the unfolded protein response (UPR; [11]). Another report suggests that mutations in different CRTs affect plant growth on Ca^2+^-deficient medium [26]. In addition, several recent studies propose that AtCRT3 is necessary for the folding of the elf18 responsive EF-Tu receptor (EFR) associated with Pathogen-Associated Molecular Patterns (PAMPs) in plants [27]; [28]. The same CRT (AtCRT3) was also recently shown to engage in the folding of a defective brassinosteroid receptor BRI-9 [29]. The latter recognition was specific for AtCRT3, and not for the other two Arabidopsis CRT isoforms.

Through expression of AtCRT1a and CRT3 in CRT-deficient mouse fibroblasts we provide evidence that the two CRT sub-groups have retained certain, but not all, CRT functions. *In planta* analyses of the family members, including gene expression, protein localization and mutant analysis, revealed that the members of the two sub-groups have acquired specialized functions, and appear to act in different biological contexts.

## Results

### Expression of different *CRT* family members in Arabidopsis

**Figure 1:**
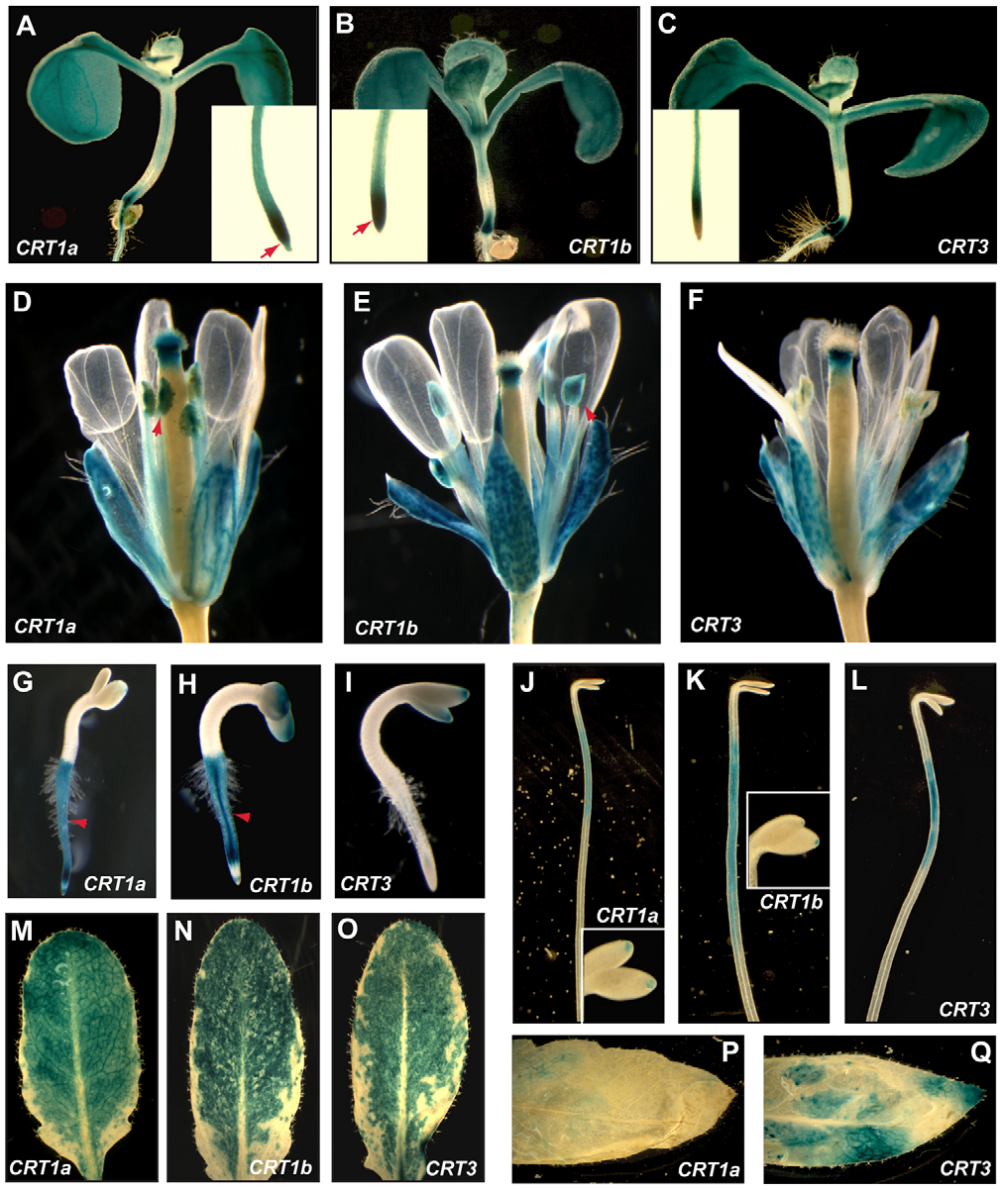
Expression of *AtCRT1a*, *CRT1b* and *CRT3*. Expression patterns of the *AtCRT1a*, *CRT1b*, and *CRT3* genes assessed through promoter:GUS constructs. The GUS expression is indicated by blue colour. **A** to **C**. Six-day-old seedlings expressing GUS-constructs for *AtCRT1a* (**A**), *CRT1b* (**B**), and *CRT3* (**C**). Root tip images for the different lines are represented as inserts. Arrows indicate expression in the root tips. **D** to **F**. Floral tissues from five week-old plants expressing GUS-constructs for *AtCRT1a* (**D**), *CRT1b* (**E**), and *CRT3* (**F**). Arrows indicate GUS activity in the pollen. **G** to **I**. Two-day-old seedlings expressing GUS-constructs for *AtCRT1a* (**G**), *CRT1b* (**H**), and *CRT3* (**I**). Arrows indicate GUS activity in the roots. **J** to **L**. Etiolated six-day-old seedlings expressing GUS-constructs for *AtCRT1a* (**J**), *CRT1b* (**K**), and *CRT3* (**L**). Inserts indicate GUS expression in the cotyledons. **M** to **O**. Rosette leaves from four-week-old plants expressing GUS-constructs for *AtCRT1a* (**M**), *CRT1b* (**N**), and *CRT3* (**O**). **P** and **Q**. Senescing leaves (from eight-week-old plants) expressing GUS-constructs for *AtCRT1a* (**P**), and *CRT3* (**Q**).

Arabidopsis seedlings lacking AtCRT1a exhibit lessened seedling growth in response to tunicamycin treatment [11]. One reason for this could be that other CRT family members are functionally redundant with AtCRT1a, i.e. in the absence of AtCRT1a the other CRTs may be sufficient to maintain CRT activity. In agreement with this, the two closely related CRT homologs *AtCRT1a* and *CRT1b* are co-expressed over approximately 2,000 publicly available microarray datasets (Figure S1A). Detailed semi-quantitative RT-PCR further revealed that *AtCRT1a* is expressed at moderate, or high levels in all major tissues investigated (Figure S1B). This is similar to what has previously been shown for *AtCRT1b* [11]. The third *CRT* family member, *AtCRT3*, is also expressed in most of the investigated tissues, with an enhanced expression in senescing leaves (Figure S1B) To examine the expression in more detail we generated *AtCRT1a*, *CRT1b* and *CRT3* promoter fusion constructs with the reporter gene β-glucuronidase (GUS), and introduced these into Arabidopsis plants. Three independent lines tested of both the *AtCRT1a* and *CRT1b* promoter constructs displayed almost identical expression patterns throughout different tissue- and cell types (Figure 1), consistent with the transcriptional coordination described above (Figure S1A). For example, these lines exhibited high GUS activity in the root tip, and expanding root cells, and were also active in rosette leaves, floral tissues including pollen, and in expanding cotyledons (Figure 1). The *AtCRT3* expression was similarly confined to expanding cells, and rosette leaves, but was absent from the root tip, and from mature pollen (Figures 1C and F). Interestingly, two-day-old seedlings were stained heavily for *AtCRT1a* and *CRT1b* promoter activity, but GUS activity in the *AtCRT3* reporter lines was virtually absent from such seedlings (Compare Figures 1G to I). In addition, whereas *AtCRT3* showed GUS activity in senescing leaves, that of *AtCRT1a* and *CRT1b* (not shown) was much reduced (compare Figures 1P and Q).

### Activation of *CRT* expression

**Figure 2:**
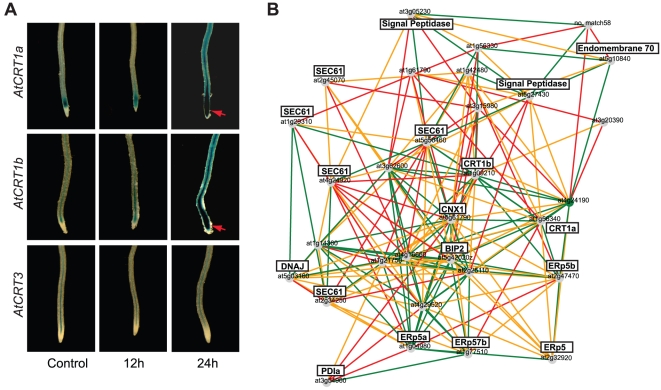
Stress induction of *AtCRT1a*, *CRT1b*, and *CRT3*. **A**. Tunicamycin induction (10 µg/ml tunicamycin) of *AtCRT1a*, *CRT1b*, and *CRT3* expression assessed by promoter:GUS constructs in five-day-old control seedlings and in seedlings treated with tunicamycin for 12 and 24 h, respectively. The GUS expression is indicated by blue colour, and by arrows. **B.** Co-expression network for *AtCRT1a* and *CRT1b* using the AraGenNet at http://aranet.mpimp-golm.mpg.de/aranet/ [44]. Brief annotations of genes are indicated in black boxes. Different colored edges indicate strength of transcriptional coordination. Green; mutual rank ≤10, Orange; mutual rank ≤20, Red; mutual rank ≤30. Low mutual rank indicates stronger co-expression relationships.

To determine whether the different *CRT* promoters may be activated by external cues we grew three independent *AtCRT1a*, *CRT1b*, and *CRT3* GUS lines on plates for three days in light. We then transferred the seedlings to microscope slides that were coated with MS medium, and grew them for two additional days. The slides were subsequently transferred to containers for exposure to different inductive treatments. We incubated the seedlings for three, 12 and 24 h in 150 mM glucose, 150 mM sucrose, 150 mM mannitol, 10 µg/ml tunicamycin, 10 mM DTT, or 250 µM salicylic acid. We chose these treatments based on publicly available microarray datasets, and on the chaperone related functions of CRTs [12]. Both the *AtCRT1a* and *CRT1b* promoters showed strong signals at 12, and 24 h after tunicamycin treatment (Figure 2A). In contrast, the activity of the *AtCRT3* promoter was not noticeably increased in response to the same treatment (Figure 2A). These data are in agreement with previous data obtained from northern blotting [24], or from microarray analyses [30]. We did not observe any major changes in the activation or repression of the *CRT* promoters in response to any of the other stresses (data not shown). The strong activation of the *AtCRT1a* and *CRT1b* promoters in response to tunicamycin is indicative of an association to tunicamycin-induced ER stress, or the UPR [11]; [13]. In animal cells, tunicamycin-mediated inhibition of N-linked glycosylation in the ER leads to the up-regulation of a battery of ER chaperones, including calnexins (CNXs), BiPs, and protein disulfide isomerases (PDIs; [31]). In agreement with this, *AtCRT1a* and *CRT1b* are closely co-expressed with other typical ER chaperone genes, including *CNXs*, *PDIs* and *BiPs* (Figure 2B; [32]), indicating that these CRTs may act as part of an ER chaperone network in Arabidopsis.

### Tissue- and cell specific distribution of CRTs in Arabidopsis

**Figure 3:**
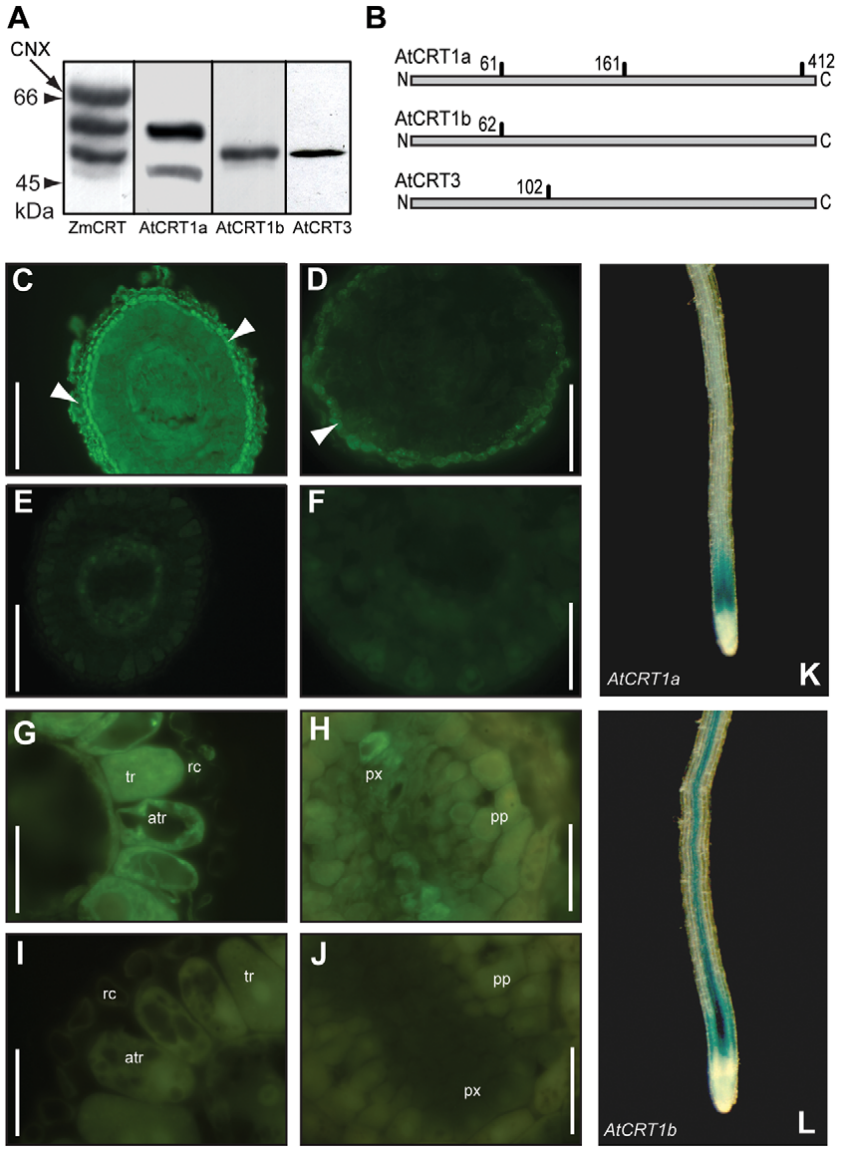
Immuno-fluorescence of AtCRT1a, and CRT1b in Arabidopsis roots. **A.** Western blot of microsomal membrane fractions from Arabidopsis cell suspensions using antibodies against a full-length maize CRT [33], and against AtCRT1a, CRT1b, and CRT3 peptides, respectively. A putative band for CNX recognized by the full-length maize CRT antibody is indicated by arrow. Twenty µg total protein was loaded per lane. **B.** Schematic model depicting potential N-linked glycosylation sites in respective Arabidopsis CRT. **C** to **F.** Root sections from 14-day-old Arabidopsis seedlings corresponding to the early elongation zone, probed with AtCRT1a, and CRT1b antibodies. **C** and **D**. Root sections probed with specific peptide antibodies for AtCRT1a (**C**), and AtCRT1b (**D**), and with pre-immune sera used as control (**E** and **F**). Scale bars = 70 µm. White arrows indicate root cap. **G** to **J**, Root sections from 14-day-old Arabidopsis seedlings corresponding to the upper elongation zone, probed with AtCRT1b antibodies (**G** and **H**), and with pre-immune sera used as control (**I** and **J**). atr; atrichoblasts, tr; trichoblasts, px; protoxylem, pp; protophloem, rc; root cap. Scale bars = 25 µm. **K** and **L**. *AtCRT1a*, and *CRT1b* expression in roots in six-day-old seedlings as assessed by GUS activity (blue colour).

To assess the distribution of the CRT proteins at the tissue- and cellular level we generated specific antibodies for the different family members. We used as antigens peptides derived from their C-terminal regions, which are flexible in structure and variable in sequence between family members. We verified the specificity of the antibodies in western blot analyses (Figures 3A). An antibody raised against a full-length maize CRT recognizes three distinct bands in typical microsomal fractions from either cell suspensions (not shown), or Arabidopsis leaves (Figure 3A; [8]). The size differences might reflect differential N-glycosylation states between the different CRTs, i.e. the AtCRT1a is predicted to have three potential N-linked glycosylation sites whereas AtCRT1b and CRT3 only contains one predicted N-linked glycosylation site each (Figure 3B; [11]). Consistent with this, the AtCRT1a antibody recognized a band that is of similar apparent molecular weight to the upper CRT band recognized by the maize antibody, and which has previously been shown to be absent in *Atcrt1a* mutant plants [11]. The AtCRT1b and CRT3 antibodies did, on the other hand, recognize bands with lower apparent molecular weights (Figure 3A). The different bands recognized by the peptide antibodies all correspond well with CRT bands recognized by the maize CRT antibody (Figure 3A).

After affinity purification we used the different CRT peptide antibodies to probe sections of Arabidopsis roots to reveal the distribution of the individual CRTs in different cell types. All the CRT family members appeared to be abundantly expressed in roots (Figure 1). The AtCRT1a antibody revealed an even distribution of this protein in most cells throughout the early elongation zone in young roots, and a high level accumulation in the root cap (Figure 3C). In equivalent sections incubated with the AtCRT1b antibody, fluorescence predominantly appeared in the outermost root cap (Figure 3D). In addition, AtCRT1b, but not CRT1a, labeling was evident in epidermal cells, both in trichoblasts and atrichoblasts, and in developing xylem cells, in sections corresponding to the late elongation zone (Figures 3G and H). These results are in agreement with GUS activity in promoter::reporter lines for the two genes, which showed strong GUS activity in the early elongation cells for *AtCRT1a*, and a slightly later onset for *AtCRT1b*, which was also active in the vascular tissues (Figures 3K and L). Furthermore, several of the trichoblasts and atrichoblasts showed immuno-fluorescence signals that appeared dotted (data not shown), possibly indicating ER-localization. We did not observe any immuno-fluorescence signal when probing with AtCRT3 antibodies, indicating that the protein accumulates at low levels, or that the epitope is masked *in situ*. No signals were obtained when probed with the respective pre-immune sera (Figures 3E, F, I and J), nor in *Atcrt1a* and *crt1b* mutants probed with AtCRT1a and CRT1b antibodies, respectively (data not shown).

### Subcellular localization of AtCRT1a, CRT1b, and CRT3

**Figure 4:**
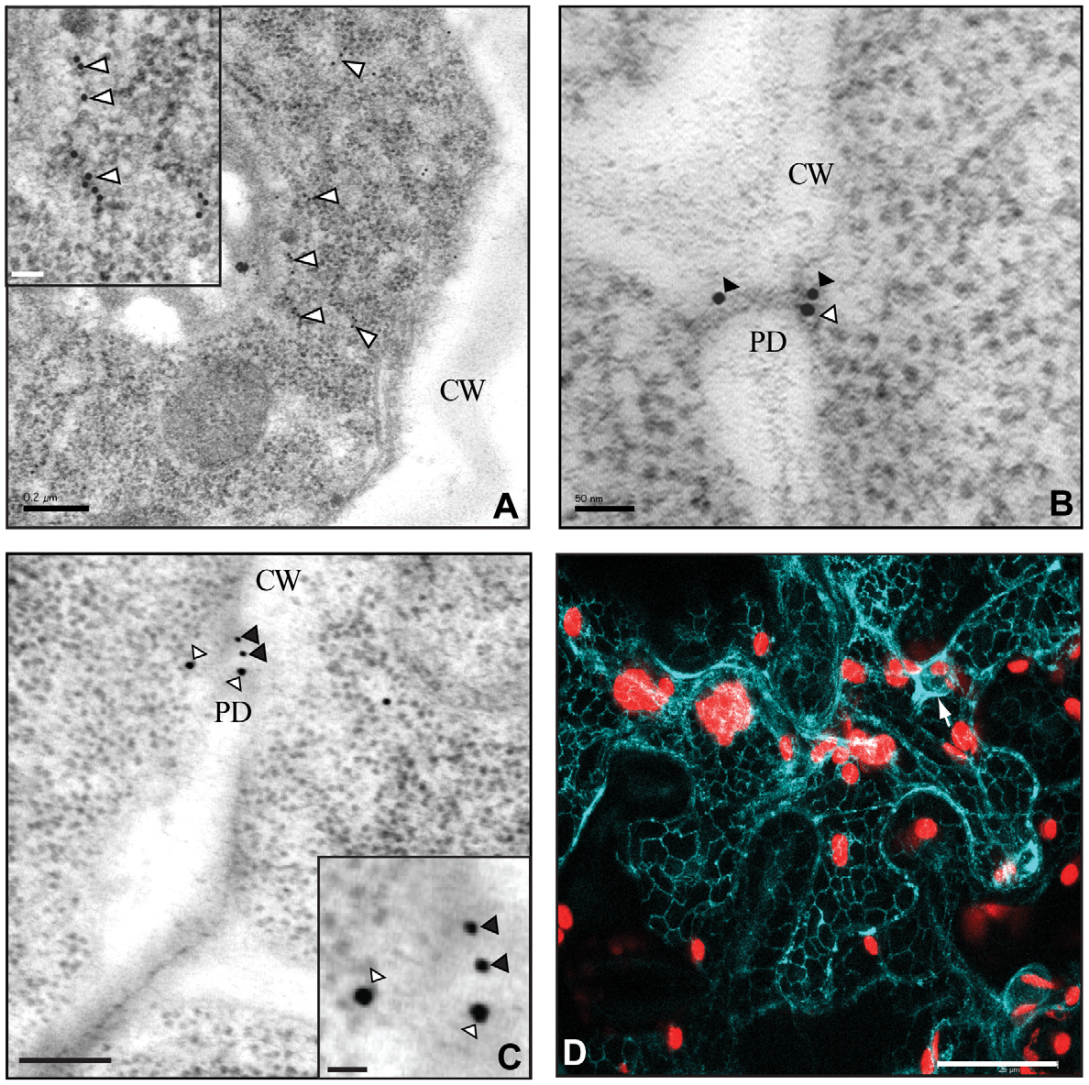
Subcellular localization of AtCRT1a, CRT1b, and CRT3. Root sections of Arabidopsis incubated with AtCRT1b (**A** and **B**), and CRT1a (**C**) peptide antibodies. **A**. AtCRT1b antibody labeling is indicated by white arrowheads. Scale bar = 0.2 µm. Insert show enlarged area of immunogold labeled area. Scale bar = 50 nm. **B**. The AtCRT1b and callose antibody labeling are indicated by white, and black arrowheads, respectively. Scale bar = 50 nm. **C**. AtCRT1a and callose antibody labeling is indicated by white, and black arrowheads, respectively. Scale bar = 100 nm. PD; Plasmodesmata, CW; Cell wall. Insert show enlarged area of immunogold labeled area; white arrow heads CRT1a, and black arrow heads Callose. Scale bar = 20 nm **D**. Transient expression of *AtCRT3* tagged with a CFP in tobacco leaves. Fluorescence is indicated in light-blue, and chloroplasts in red. Scale bar = 500 nm. Arrow indicates nuclear envelope.

The immuno-fluorescence data suggest that the two CRTs, AtCRT1a and CRT1b, accumulate in partially overlapping but distinct cell types. To determine whether there are also differences in the subcellular distribution we utilized immuno-cytochemical techniques together with transmission electron microscopy (TEM). The AtCRT1b antibody recognized epitopes that appeared associated with the ER, e.g. ribosome enriched segments, in Arabidopsis root sections from the early elongation zone (Figure 4A). No labeling was observed in an Atcrt1b mutant under the same conditions (data not shown). In addition, we also found AtCRT1b epitopes in structures resembling plasmodesmata (Figure 4B). To confirm the plasmodesmata localization we co-incubated the sections with the AtCRT1b antibody raised in rabbits, and a callose (β-1, 3–glucan) antibody raised in mice. Consistent with localization of AtCRT1b to the plasmodesmata the callose antibody labeled regions in close vicinity to the AtCRT1b labeling (Figure 4B). Similarly, we observed AtCRT1a associated with the plasmodesmata (Figure 4C), and also with intracellular compartments reminiscent of the ER (data not shown). However, labeling by the AtCRT1a antibodies appeared less dense than by the AtCRT1b antibody, possibly because the binding epitope was obscured, or perhaps due to lower levels of AtCRT1a in the analyzed cells.

Many attempts were made to visualize the AtCRT3 by immuno-gold labeling but, analogous to the immuno-labeling attempts, we were unable to obtain any signals above the pre-immune serum control. Since contrasting localization data has been presented for AtCRT3, i.e. ER (27) and cytoplasm or nucleus (25), we argued that it would be important to consolidate the subcellular location of the protein. We therefore generated an AtCRT3 fusion with the fluorescent marker CFP (cyano fluorescent protein) under the control of a 35S promoter. We inserted the CFP seven amino acids upstream of the C terminus to minimize a possible influence on the activity of its putative ER-retention signal (HDEL). We subsequently transiently transformed the construct into tobacco leaves, and visualized the CFP signal under a confocal microscope (Figure 4D). The CFP signal displayed a reticular network, reminiscent of the ER. In addition, the signal also clearly outlined the nucleus, possibly representing the nuclear envelope (Figure 4D). These data are consistent with the predicted sub-cellular localization of CRTs, i.e. in the ER [12], and with the fact that AtCRT3 holds an ER retention signal in its far C terminus.

### *AtCRT3* expression in *crt^−/−^* mouse fibroblasts

**Figure 5:**
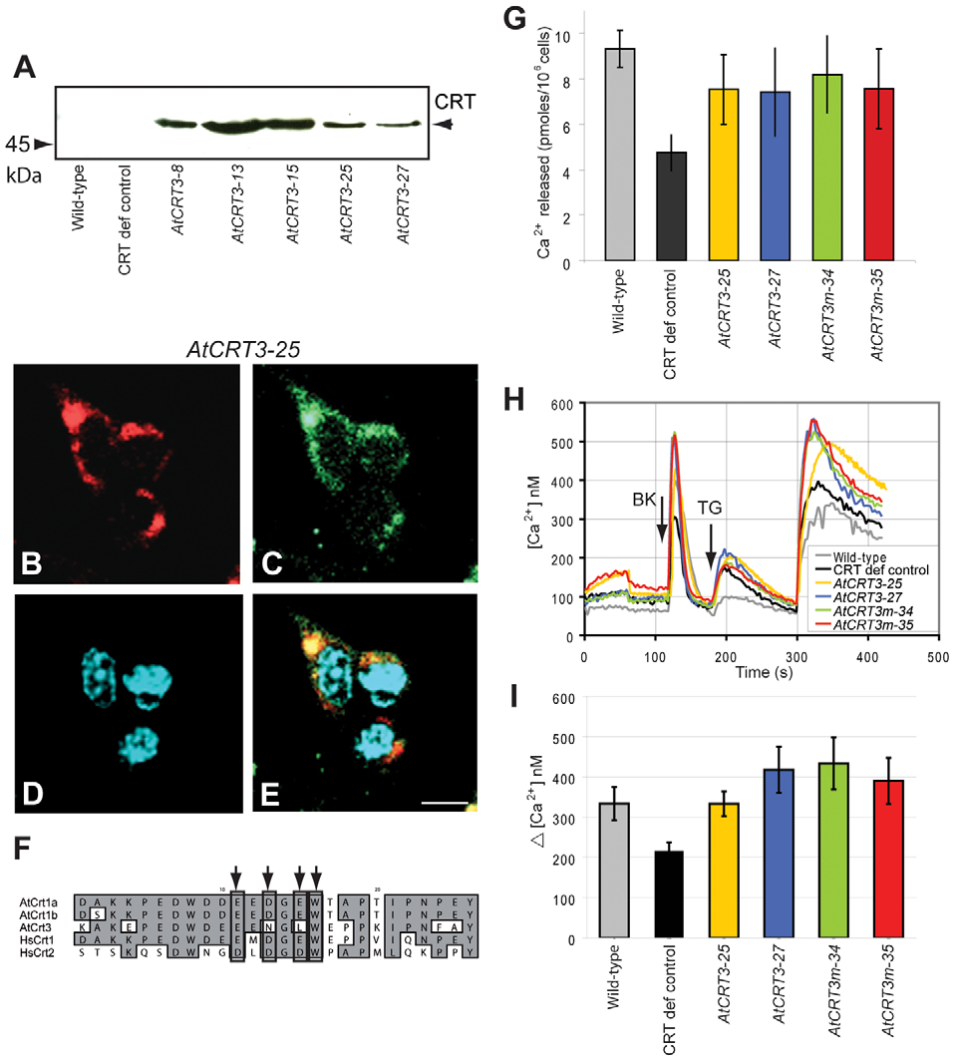
AtCRT3 complementation of *crt^−/−^* mouse fibroblasts. **A**. Immunoblot analysis of AtCRT3 expressed in *crt^−/−^* mouse fibroblasts. The PVDF membranes were probed with anti-HA-tag antibodies. Twenty µg total protein was loaded per lane. **B** to **E**. Immuno-labeling of AtCRT3 expressed in CRT-deficient mouse cells (line *AtCRT3–25*) with anti-HA-tag (AtCRT3; **B**), anti-PDI (ER-marker; **C**), and DAPI (nuclear-marker; **D**). **E**. Overlay, the yellow color indicates identical localization of ER-marker, and AtCRT3. Scale bar = 25 µm. **F**. Comparison of amino acid sequences of AtCRT1a, CRT1b and CRT3 and human CRT1 and CRT2 (GenBank accession no. AAC49695, AAK74014, AAC49697, AAA51916, NP_659483, respectively). The black rectangles represent the amino acids residues important for CRT function. **G**. ER Ca^2+^ content measurements in different mouse fibroblast lines. Total cellular Ca^2+^ content was determined by incubation with 55 µCi ^45^Ca^2+^ followed by addition of thapsigargin. **H**. Measurements of bradykinin (BK)-induced Ca^2+^ release in different mouse fibroblast lines. Mouse fibroblasts were loaded with the fluorescent Ca^2+^ indicator Fura-2 followed by stimulation with BK. Typical traces showing cytosolic Ca^2+^ levels before and after addition of BK, and thapsigargin (TG). The experiments were carried out using a Ca^2+^-free medium. **I**. ΔCa^2+^ after addition of BK from (**H**). Wild-type; Mouse fibroblast containing CRT, CRT def control; CRT-deficient cell line, *AtCRT3* and *AtCRT3m*; CRT-deficient cell lines complemented with *AtCRT3* constructs. Results represent the average ± SE of three independent experiments.

We have previously shown that AtCRT1a restores CRT deficiencies in mouse fibroblasts [11]. To test whether also AtCRT3 can complement the CRT-deficient mouse cells we expressed the *AtCRT3* fused to a HA-tag in these cells. Five stable transgenic cell lines were established, and expression was confirmed using immuno-blotting with a HA-tag antibody (Figure 5A). A single band at 60 kDa was detected in all *AtCRT3* lines, while no bands were present in the CRT-deficient control lines. To confirm that the recombinant AtCRT3 was located to the ER, immuno-labeling using the HA-tag antibody was performed (Figure 5B, red color). All AtCRT3 transgenic cell lines showed ER-localization, which was confirmed by co-localization with an antibody against the ER marker PDI (Figures. 5C and E). These data support the ER localization of the CFP-tagged CRT3 in tobacco leaves (Figure 4D).

Most animal CRTs do not hold N-linked glycans (Frickel and Ellgaard, 2003). To test whether plant CRTs, i.e. the AtCRT3 and CRT1a [11], undergo N-linked glycosylation when expressed in the mouse cells we treated lysates derived from mouse cells, expressing either *AtCRT1a* or *CRT3*, with the glycosidase PNGase F that removes N-linked glycans (Figure S2A). The glycosidase treatment of cell lysates decreased the apparent molecular weights of the plant CRTs on immunoblots (Figure S2A), suggesting that the AtCRT1a and CRT3 may acquire N-linked glycans when expressed in the mouse cells.

### Ca^2+^-storage, and agonist-induced capacities are restored by expressing *AtCRT3* in *crt^−/−^* mouse fibroblasts

AtCRT3 contains a smaller number of negatively charged amino acids in its C terminus compared to AtCRT1a and CRT1b, and most animal CRTs, indicating a lower Ca^2+^-holding ability of the protein [33]. Notably, most amino acids that are important for chaperone functions in animal CRTs are conserved in AtCRT1a and CRT1b, but not in AtCRT3 (Figure 5F; [34]). For example, the amino acids corresponding to Asp^258^ and Glu^260^ in animal CRT (numbers referred to human CRT1) that are necessary for chaperone activities and ERp57 interactions [34] have been changed to Asn^268^ and Leu^270^ in AtCRT3 (Figure 5F). Similar changes are also seen in CRT3 orthologs in maize and rice (data not shown). To assess whether these changes may distinguish AtCRT3 functions from that of typical CRTs, we measured the ER Ca^2+^-holding potential and the responsiveness to BK of the AtCRT3 complemented *crt^−/−^* mouse fibroblasts. These fibroblasts were cultured in the presence of ^45^Ca^2+^ for 50 h, as described in [11]. Addition of the ER Ca^2+^-ATPase blocker thapsigargin caused a significant increase (approximately 1.5-fold) in Ca^2+^-release in *AtCRT3* expressing cells compared with the CRT-deficient control cells (Figure 5G).

We further measured the BK-induced Ca^2+^-release in the AtCRT3-transfected fibroblasts, via the fluorescent Ca^2+^ indicator Fura2 [3]. The basal Ca^2+^ level in the cytosol before addition of BK was estimated to be ∼100 nM for all cell lines (Figure 5H). Addition of BK to the cells resulted in a rapid release of Ca^2+^ in all lines tested (Figure 5H). However, the release from the mouse control, and *AtCRT3* expressing cells were approximately 1.5- to 2-fold higher as compared to that from the CRT-deficient control cells (Figure 5H). In addition, the amounts of Ca^2+^ release in the mouse wild-type, and *AtCRT3* expressing lines were similar, indicating that AtCRT3 restored the CRT deficiencies in the *crt^−/−^* fibroblasts (Figure 5I). These data are similar to data obtained from *AtCRT1a* expressing *crt^−/−^* fibroblasts [11], and show that AtCRT3 holds basic CRT functions.

### Mutations in *AtCRT3* do not enhance CRT activities in *crt^−/−^* mouse fibroblasts

In spite of the alteration of the amino acids Asp^258^ and Glu^260^ conserved in animal CRTs to Asn^268^ and Leu^270^ in AtCRT3, AtCRT3 restored the ER Ca^2+^-holding, and BK-induced release of Ca^2+^ in the CRT-deficient mouse cells. To assure that this is the case we introduced these amino acids, essential for ERp57 interaction (Asp and Glu), to the positions of Asn^268^ and Leu^270^ in AtCRT3, respectively, and then generated five transgenic mouse cells expressing this AtCRT3 variant, referred to as AtCRT3m. We verified that the expression and localization of AtCRT3m was similar to that of AtCRT3 (Figures S2B to F). The thapsigargin-sensitive ER Ca^2+^ content in the *crt^−/−^* mouse fibroblasts expressing *AtCRT3m* was very similar to the thapsigargin sensitive ER Ca^2+^ storage capacity of the *AtCRT3* expressing cells (Figure 5G). Similarly, no significant differences between the AtCRT3 and CRT3m were detected when we assessed the BK-induced Ca^2+^ releases (Figures 5H, I, and S3). These data suggest that the introduction of the Asp^258^ and Glu^260^ conserved in most CRTs, at corresponding sites to Asn^268^ and Leu^270^ in AtCRT3 do not affect localization of the protein, the ability to retain Ca^2+^ in the ER, nor the capability to sustain BK-induced Ca^2+^-release from the ER in the mouse fibroblast system.

### Expression of *AtCRT1a* and *CRT3* in CRT-deficient mouse cells do not restore cell adhesion deficiencies

**Figure 6:**
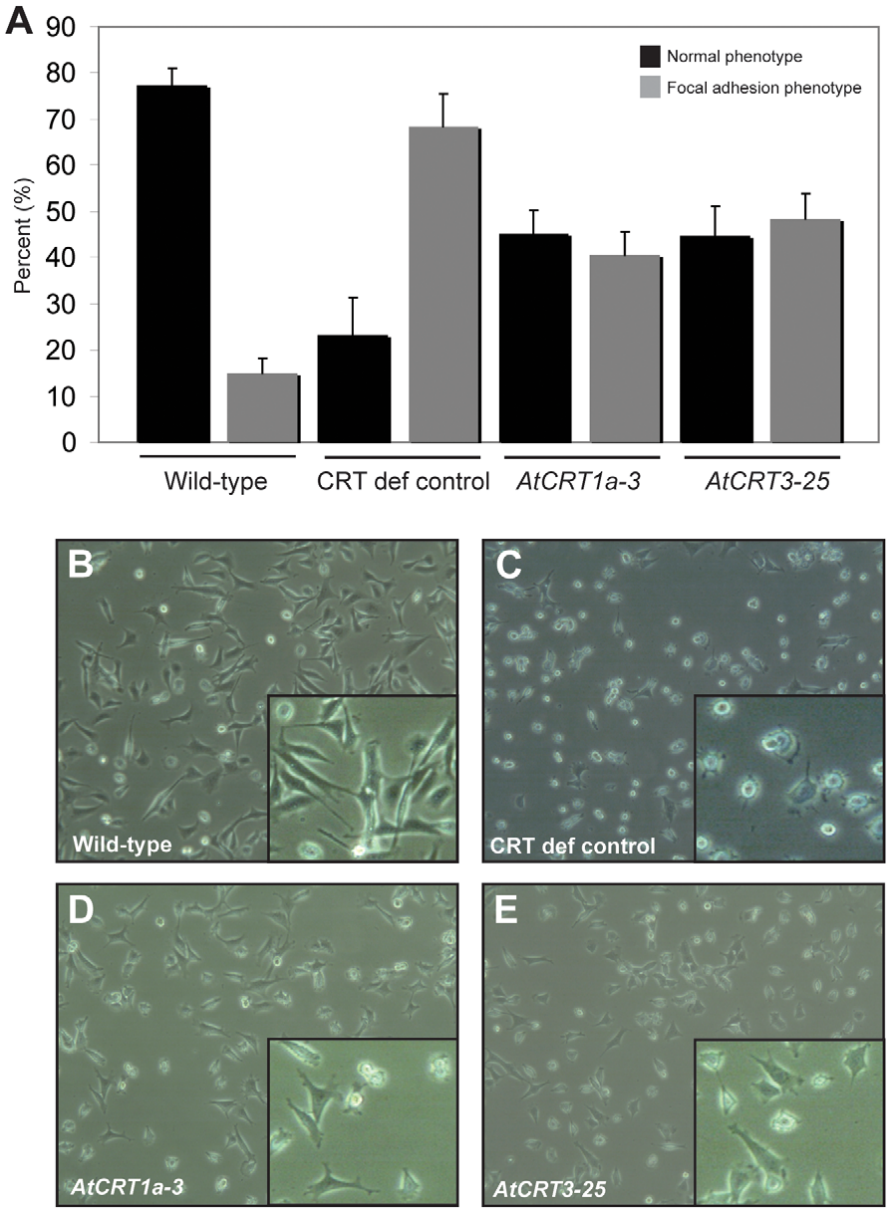
Cell adhesion in *AtCRT1a* and *CRT3* expressing *crt^−/−^* mouse fibroblasts. **A**. Relative value of normal, and focal contact, phenotype with regards to the total number of the cells of a 25 mm cell culture dish. **B** to **E**. Phase contrast images of various cell lines after 16 h growth. Inserts display magnified part of image. Wild-type; Mouse fibroblast containing CRT, CRT def control; CRT-deficient cell line, *AtCRT1a* and *AtCRT3*; CRT-deficient cell lines complemented with *AtCRT1a* and *AtCRT3* constructs, respectively. Results represent the average ± SE of three independent experiments.

In addition to compromised ER Ca^2+^ levels and BK-induced Ca^2+^ releases, the crt^−/−^ fibroblasts also exhibit a lower degree of cell-substratum adhesion [4]. To test whether expression of *AtCRT1a* or *CRT3* in the crt^−/−^ mouse fibroblasts have regained their adhesive potency we determined the cell shape, which is conventionally examined to assess cell spreading, and therefore the level of cell adhesion [4]. Figures 6A and B clearly show that wild-type cells, which contain CRT, readily adhered to the surface. In contrast, the CRT-deficient cells did not (Figures 6A and C). Interestingly, the *AtCRT1a* and *CRT3* expressing cell lines that restored the ER Ca^2+^ content, and the BK-induced Ca^2+^ release from the ER only partially restored the cell adhesiveness (Figures 6A, D and E). Approximately, 50% of the *AtCRT1a* and *CRT3* expressing cells showed cell spreading, whereas wild-type cells typically had above 75% cell spreading (Figure 6A). Thus the level of cell adhesion is not fully restored by the plant CRTs. This suggests that restoration of the ER Ca^2+^ content alone is not sufficient to fully restore cell adhesion activity.

### Mutations in *AtCRT1b* result in altered tunicamycin responsiveness, and in retarded seedling growth

**Figure 7:**
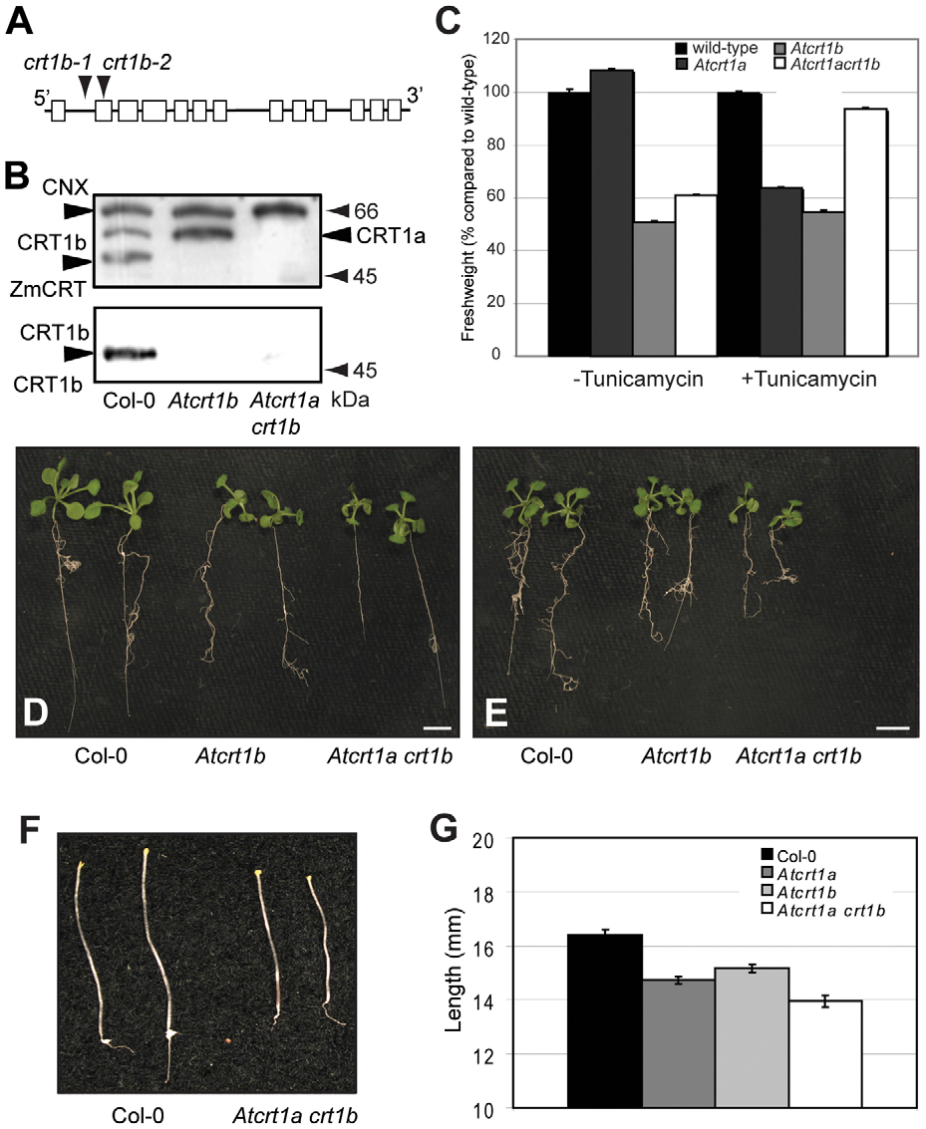
*Atcrt1b* single, and *Atcrt1a crt1b* double mutant characterization. **A.** Schematic representation of approximate localization of the T-DNA lines for *Atcrt1b*. **B.** Western blot of microsomal proteins of *Atcrt1b-1* single, and *Atcrt1a crt1b* double mutants using maize CRT antibodies (1∶10 000; upper panel), and AtCRT1b antibodies (1∶2000; lower panel). Twenty µg total protein was loaded per lane. **C** to **E.** Images (**D** and **E**), and fresh weight measurements (**C**) of 14-day-old seedlings grown on control MS media (**D**), and MS media supplemented with 0.1 µg/ml tunicamycin (**E**), respectively. **C.** Values are indicated as percent compared to wild-type on control media (left), and to wild-type on tunicamycin supplied media (right). From left, wt, *Atcrt1b* single, and *Atcrt1a crt1b* double mutants. Scale bars = 5 mm. SE = standard error (n = 20). **F** and **G**. Image (**F**), and hypocotyl length measurements (**G**) of 6-day-old etiolated seedlings grown on MS media. From left, wt, and *Atcrt1a crt1b* double mutants. SE = standard error (n = 20).

We previously described wild-type like growth in *Atcrt1a* mutant plants under normal growth conditions [11]. However, *Atcrt1a* mutants exhibited an increased sensitivity to the UPR-inducing agent tunicamycin. This relatively weak phenotype, i.e. lessened seedling growth, in response to tunicamycin may be due to functional compensation by AtCRT1b that is present in the mutant. To test this, we isolated two independent *Atcrt1b* mutant lines that appear to be null mutants for the encoded protein as assessed by western blotting (Figures 7A and B). The western analysis also revealed that the AtCRT1b band migrated to an apparent lower molecular weight (Figure 7B), consistent with the notion that AtCRT1b may hold lower levels of glycans compared to AtCRT1a.

Under normal growth conditions mature *Atcrt1b* mutant plants did not display any significant phenotypes compared to wild-type control plants. However, both light-grown and etiolated seedlings were significantly smaller compared to wild-type seedlings (Figures 7C to G). Interestingly, *Atcrt1b* seedlings exhibited a relatively lower sensitivity to tunicamycin compared to wild-type control seedlings (Figures 7C and E). These data may suggest that loss of AtCRT1b constitutively triggers a stress similar to UPR in the seedlings, or that the perception of this type of stress is altered in the mutant seedlings.

### *Atcrt1a crt1b* double mutants are impaired in hypocotyl expansion

To assess whether the relatively mild phenotypes for *Atcrt1a* and *crt1b* are due to functional redundancy between the two homologs we generated *Atcrt1a crt1b* double mutants. The absence of both proteins was confirmed in western blot analyses (Figure 7B). Although we did not observe any obvious phenotypes in mature plants, the double mutant seedlings were significantly smaller compared to the wild-type line (Figures 7C and D), and to the *Atcrt1a* mutant parent line (Figure 7C). However, there was no major size difference between the *Atcrt1b* mutant and the *Atcrt1a crt1b* double mutant seedlings grown in light (Figure 7C). These data suggest a predominant role of AtCRT1b over AtCRT1a in light-grown seedlings. However, when grown in the dark the *Atcrt1a crt1b* double mutant did exhibit an additive phenotype compared to the *Atcrt1a* and *crt1b* single mutant parent lines (Figures 7F and G), suggesting that both AtCRT1a and CRT1b contribute to etiolated seedling growth. The *Atcrt1a crt1b* seedlings displayed lower sensitivity to tunicamycin-dependent growth retardation as compared to wild-type seedlings (Figures 7C to E) indicating that they either are under constitutive ER stress, or that the perception of such stress is different in the mutants. Consistent with this hypothesis both the *Atcrt1b* and the *Atcrt1a crt1b* mutants were two- to four-fold less sensitive to tunicamycin compared to wild-type control seedlings (data not shown), corroborating that the mutants may be primed for ER stress prior to the tunicamycin treatment.

**Figure 8:**
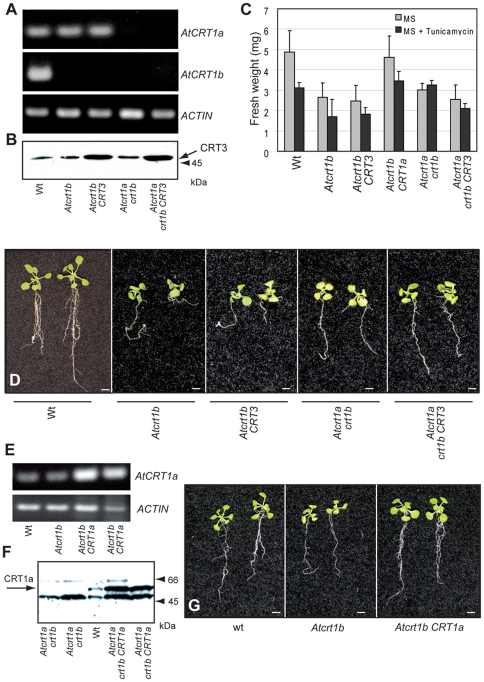
Complementation of the *Atcrt1b* single, and *Atcrt1a crt1b* double mutants with AtCRT1a, or CRT3. **A.** Expression of *AtCRT1a*, and *AtCRT1b* in the different mutant combinations assessed by semi-quantitative RT-PCR. *ACTIN* was used as control. **B.** Western blot using the peptide antibodies against AtCRT3 to assess production of AtCRT3 in the 35S:AtCRT3 transformed mutant lines. Twenty µg total protein was loaded per lane. **C** to **F**. Assessment of complementation of *Atcrt1b* single and *Atcrt1a crt1b* double mutants by *AtCRT1a* or *CRT3* under the control of a 35S promoter, respectively. **C**. Graph of fresh-weight for 14-day-old wt, *Atcrt1b* single, and *Atcrt1a crt1b* double mutant seedlings, and of mutant seedlings over-expressing *AtCRT1a* or *CRT3*, respectively, grown on MS medium. The graph depicts one out of three experimental repeats. **D**. Images of typical 14-day-old wt, *Atcrt1b* single, and *Atcrt1a crt1b* double mutant seedlings, and of mutant seedlings over-expressing *AtCRT3* grown on MS medium. Scale bars = 5 mm. **E**. Expression of *AtCRT1a* in *Atcrt1b*, or in *Atcrt1b* transformed with *AtCRT1a* under a 35S promoter. *ACTIN* was used as control. **F.** Western blot using the maize CRT antibody to assess production of AtCRT1a in the 35S:AtCRT1a transformed *Atcrt1a crt1b* double mutant lines. Twenty µg total protein was loaded per lane. **G**. Images of typical wt, and of *Atcrt1b* mutants that were complemented with *AtCRT1a* under the control of a 35S promoter. Scale bars = 5 mm.

One explanation for the lower tunicamycin sensitivity in the mutant seedlings could be that they constitutively produce higher levels of different ER chaperones in the absence of AtCRT1b. However, in the case of the *Atcrt1b* single mutants, it is likely that an increased production of, for example, the tunicamycin-sensitive AtCRT1a would mask any growth phenotype. Indeed, this scenario is corroborated by the fact that AtCRT1a complemented the *Atcrt1b* growth phenotypes when over-expressed (Figure 8; see below). To assess whether the expression of ER chaperones is constitutively active in the mutants we performed real-time PCR analysis on a range of such genes, which have been shown to be induced by tunicamycin [30]. Figure S4 shows no significant differences in the basal expression levels of these chaperone-related genes in the mutants as compared to wild-type control seedlings. Although it cannot be ruled out that these putative chaperones are altered at the protein level, it is plausible that the perception of, or response to, tunicamycin is altered in *Atcrt1b* and *Atcrt1a crt1b*.

### *AtCRT1a*, but not *AtCRT3*, expression restores growth in *Atcrt1b* and *Atcrt1a crt1b* mutants

To test whether the different Arabidopsis CRTs could restore the growth defects in the *Atcrt1b* single, and *Atcrt1a crt1b* double mutants we introduced two constructs with either an *AtCRT1a* cDNA or an *AtCRT3* cDNA driven by a constitutive 35S promoter into the two mutant lines. To confirm the expression of *AtCRT1a* and *CRT3* we performed semi-quantitative RT-PCR, and western blotting, respectively (Figures 8A, B, E, F and S3B). Several independent *AtCRT1a*-transformed *Atcrt1b* mutant lines displayed an increase in *AtCRT1a* mRNA production and AtCRT1a protein accumulation (Figures 8E and F). Similarly, mutant lines transformed with *AtCRT3* displayed increased levels of AtCRT3 protein (Figures 8B and S3B). These data show that the transformed mutant lines produce higher levels of AtCRT1a and CRT3 compared to the untransformed mutant lines, respectively. AtCRT1a restored the growth to wild-type levels of the *Atcrt1b* mutant (Figures 8C and G), indicating that the AtCRT1a and CRT1b proteins are functionally redundant at least during seedling growth. In contrast, AtCRT3 did not complement the *Atcrt1b*, nor the *Atcrt1a crt1b* double mutant phenotypes (Figures 8C and D). These differences were significant (t-tests∶ t_9_ = 5.74, P = 1.3×10^−5^ for *Atcrt1b* compared to *Atcrt1b AtCRT3*), suggesting a divergent role of AtCRT3 from AtCRT1a and CRT1b.

### *Atcrt3*, but not *Atcrt1a* nor *crt1b*, mutants are impaired in elf18 perception, and signaling in Arabidopsis

**Figure 9:**
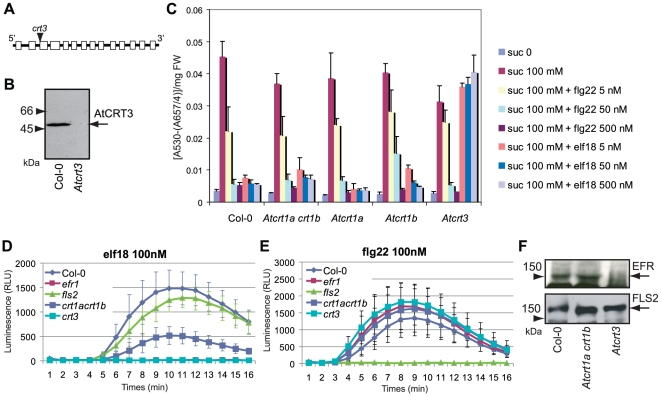
PAMP-induced responses in *Atcrt* mutants. **A.** Schematic representation of approximate localization of the T-DNA line for *Atcrt3*. **B.** Western blot of *Atcrt3* using AtCRT3 peptide antibodies (1∶2000). **C.** Anthocyanin content in 6-day-old wt and mutant seedlings was determined after incubation for three days with, or without, 100 mM sucrose, and PAMPs as indicated on the right. **D** and **E**. PAMP-induced oxidative burst in 4-week-old wt and mutant seedlings. Leaf discs derived from 4-week-old plants were treated with PAMPs as described in Saijo et al. (accepted elsewhere). **F**. Western blot analysis of 4-week-old plant leaves. Microsomal membrane fractions were produced for the different mutants, and subjected to immuno-blot analysis with the indicated antibodies. *efr1* and *fls2* indicate seedlings mutated in *EFR* and *FLS2*, respectively. Ten µg total protein was loaded per lane.

**Figure 10:**
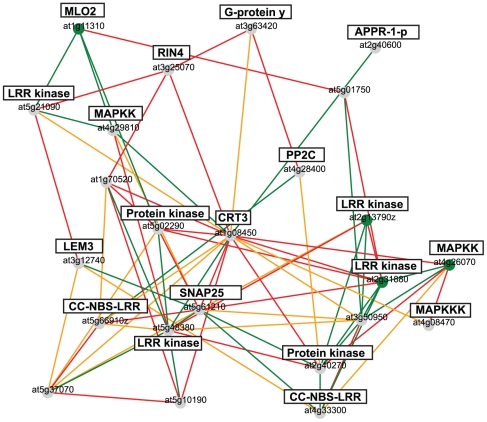
*AtCRT3* co-expression relationships. Co-expression network for *AtCRT3* using the AraGenNet at http://aranet.mpimp-golm.mpg.de/aranet/ [44]. Brief annotations of genes are indicated in black boxes. Different colored edges indicate strength of transcriptional coordination. Green; mutual rank ≤10, Orange; mutual rank ≤20, Red; mutual rank ≤30. Low mutual rank indicates stronger co-expression relationships.

To assess whether mutations in the *AtCRT3* gene affect any aspects of plant growth we screened T-DNA lines for homozygous inserts in the gene (SALK_051336). AtCRT3 protein was undetectable in homozygous mutant plants, confirming that the T-DNA insertion disrupted the expression of the gene (Figures 9A and B). No obvious growth phenotypes were evident in the mutant compared to wild-type under normal growth conditions, nor upon tunicamycin treatment. These data suggest a minor role of AtCRT3 under UPR conditions, which is consistent with undetectable *AtCRT3* activation upon tunicamycin application (Figure 2A). In addition, *AtCRT3* is not part of the closely co-expressed ER chaperone network that contains *AtCRT1a* and *CRT1b*. However, we noticed that *AtCRT3* is instead transcriptionally coordinated with several genes that are responsive to biotic and abiotic stresses (Figure 10). For example, two of the MAP kinase kinases (MAPKK), *MKK1* and *MKK2*, are closely co-expressed with *AtCRT3* (Figure 10). Both MKK1 and MKK2 appear to be part of a phosphorylation cascade that is triggered by PAMPs, such as elf18, and flg22 (For review see [35]). Consistent with this [27]; [28] recently showed AtCRT3 is necessary for elf18 perception and responses. To test whether AtCRT1a and CRT1b also participate in the responsiveness to PAMPs, we tested possible alterations in PAMP responses in the absence of these CRTs. First we grew the single-, and double mutant lines on medium containing 100 mM sucrose in the absence, or presence of elf18 or flg22 (Figure 9C). These peptides suppress anthocyanin accumulation in wild-type seedlings that are exposed to high levels of sucrose (Figure 9C). However, while no elf18-induced anthocyanin suppression was observed in *Atcrt3* seedlings (Figure 9C; [27]; [28]), both the *Atcrt1a* and *crt1b* single, and the *Atcrt1a crt1b* double mutants showed wild-type-like suppression of anthocyanin accumulation in the presence of elf18 (Figure 9C). These results are in agreement with the recent report from Li et al. [27]. The de-repression of anthocyanin accumulation in the *Atcrt3* mutants in the presence of elf18 has been attributed to the defects in the biogenesis, and stable accumulation, of the receptor-like kinase EFR that acts as the elf18 receptor (Figure 9D; [27]; [28]). Consistent with these data, we detected no significant alteration in the steady-state levels of EFR in the *Atcrt1a crt1b* double mutants compared to wild-type (Figure 9F, upper panel; Figure S3C).

The perception of PAMPs induces a transient generation of reactive oxygen species (ROS) at the outer-surface of the plasma membrane [35]. While this response is readily triggered in the *Atcrt3* mutant by flg22, elf18 failed to induce ROS in this mutant (Figures 9D and E; [27]; [28]). Interestingly, the ROS generation was also partially impaired in the *Atcrt1a crt1b* double mutants, albeit not to the same extent as in the *Atcrt3* mutants, similar to what has recently been reported in [27] (Figure 9D). These data either indicate that EF receptor outputs are not fully retained in the *Atcrt1a crt1b* double mutant, or that the production of ROS is impaired in this mutant.

## Discussion

The multi-functional protein CRT has been widely studied in animals where the protein is associated with more than 40 cellular functions [36]. Studies on CRTs in plants are less advanced, in particular in that specific roles of the individual CRT family members present in plants have, until recently, been largely unaddressed. We show that while the three different CRT family members in Arabidopsis have retained basic CRT functions, they have attained diverged functions *in planta*.

Both AtCRT1a and CRT3 restored the ER Ca^2+^ levels, and putative chaperone deficiencies in the *crt^−/−^* mouse fibroblasts. Such complementation was somewhat unexpected for AtCRT3 given significant divergence in its protein sequence that may be predicted to hamper characteristic CRT activities. For example, the protein only holds negatively charged amino acid residues in 28 of the 110 residues of the C-domain. In comparison, AtCRT1a holds 44 negatively charged amino acids in the equivalent region. These negatively charged amino acids are believed to constitute the main Ca^2+^-binding mechanism of the CRTs [33]. AtCRT1a does, similar to AtCRT3, also complement the CRT deficiencies in the mouse fibroblast system. More detailed analyses are necessary to distinguish any differences that are attributable to the different numbers of negatively charged amino acids between the two CRT isoforms. Interestingly, [29] recently showed that the C-terminal part of AtCRT3 is crucial for the protein to retain the defective BRI1-9 in the ER. These data further support an important role for the C-domain of the CRTs for the divergent functions.

Surprisingly, while the ER Ca^2+^ homeostasis and the putative chaperone functions were restored in the CRT-deficient mouse fibroblasts by expression of either *AtCRT1a* or *CRT3*, other phenotypes were only partially complemented. For example, the reduced cell adhesiveness associated with the CRT-deficient fibroblasts could not be fully rescued by either AtCRT1a or CRT3 (Figure 6). However, it is important to point out that the relative expression level of the *AtCRT1a* and *CRT3* also may influence the adhesive ability in the mouse fibroblast. While this may be the case, it is clear that the Ca^2+^ levels were restored to control levels in the *AtCRT1a* and *CRT3* expressing lines, and it is therefore unlikely that the defects in adhesion of these cells are due to a decrease in the ER Ca^2+^ content in the transfected *crt^−/−^* fibroblasts. Rather, our results suggest other CRT characteristics that are not conserved in the plant CRTs. The adhesion between the cells and substrate depends on several proteins connecting the cell surface to the cytoskeleton, such as fibronectin and vinculin, and also on tyrosine phosphorylation cascades [4]; [6]. It is possible that AtCRT1a and CRT3 are unable to properly fold some of these components, and thus fail to restore cell adhesion activity. It is also plausible that AtCRT1a and CRT3 are unable to efficiently fold different Ca^2+^-releasing components in the ER, or that AtCRT1a and CRT3 fail to interact with these components that may require a direct interaction with CRT to facilitate Ca^2+^-signaling. Such impairments could of course also impair Ca^2+^-releases and therefore cell adhesion [4]. In any case, the inability of the AtCRT1a and CRT3 to fully restore the cell adhesion phenotype in the CRT-deficient cells suggets that while some functions are conserved between the CRTs in the two kingdoms there may also be diverged characteristics that are specific to plant and animal CRTs.

Analyses of publicly available expression data, and our promoter:GUS experiments, suggest that expression of *AtCRT1a* and *CRT1b* is active in the same tissue- and cell types (Figure 1). However, immuno-fluorescence data using peptide specific antibodies clearly show that the similarity in expression only partly is mirrored on the protein level (compare Figures 1 and 3). These results suggest that while the two CRTs may be active in different cell types, certain cells may need additional CRT activity perhaps for increased protein production, or for an increased need for ER Ca^2+^ holding potential. The potential functional overlap between these CRTs was corroborated by the complementation of *AtCRT1b* mutants by AtCRT1a. *AtCRT1a* and *CRT1b* are also co-expressed with several other ER chaperones, including *CNXs*, *BiPs* and *PDIs*, and are induced by tunicamycin, a trigger of UPR in the ER [30]. Several reports show that genes that are transcriptionally coordinated tend to be functionally related (for example [37]; [38]). It is therefore tempting to speculate that the AtCRT1a and CRT1b are part of a larger network of ER chaperones, which could provide a basal folding apparatus of newly synthesized proteins in the ER.

Although our data suggest a role for AtCRT1a and CRT1b in the UPR ([11]; Figure 7), we did not see any indications for a similar function for AtCRT3. Instead, the *Atcrt3* mutant plants displayed defects in elf18 responses, which were not observed to the same extent in the *Atcrt1a crt1b* double mutant plants (Figure 9, [27]; [28]). These results are in agreement with two recent reports [27]; [28], which showed failure of *Atcrt3* mutants to repress anthocyanin accumulation in response to elf18. Interestingly, genes encoding MKK1 and MKK2 that mediate PAMP responses [39] are closely co-expressed with *AtCRT3* (Figure 10). In addition, several other immune response-related genes are also co-expressed with *AtCRT3*. These include two genes encoding CC-NBS-LRR homologs. Several family members of this class of proteins act as resistance (R) proteins and confer isolate-specific immunity [40]. Another co-expressed gene is *RIN4*, a gene required for immune-responses to phytopathogenic *Pseudomonas syringae* conditioned by the R proteins RPS2 and RPM1 (Figure 10; [41]). These data are suggestive of a role for AtCRT3 in immune responses.

Both EFR and Flagellin-sensing 2 (FLS2) contain multiple putative N-glycosylation sites in their LRR domains [42]. This domain is predicted to be exposed to the ER lumen during protein folding, and maturation. It is therefore tempting to speculate that AtCRT3 recognizes the N-linked glycans attached to the unfolded EFR, thereby becoming engaged in the folding of the receptor. A similar recognition does not seem to occur for the FLS2 (Figure 9), or if it does, the ER holds other agents that are sufficient to facilitate correct folding of the FLS2 in the absence of ACRT3. Since AtCRT1a and CRT1b do not seem to be predominantly involved in the folding of the EFR (Figures 9; [27]), it is apparent that the different subgroups of CRTs have developed substrate specificity. This notion was further corroborated by the inability of *AtCRT3* to complement the *Atcrt1b*, and the *Atcrt1a crt1b* plants even when over-expressed (Figure 8). In contrast, over-expression of *AtCRT1a* complemented *Atcrt1b* plants. Given that both the AtCRT1a and CRT3 restored the ER Ca^2+^ levels in the CRT-deficient mouse fibroblasts (Figure 5; [11]), we assume that the Ca^2+^ storage activity is similar between these two types of plant CRTs. We therefore propose that AtCRT1a and CRT3 mainly differ in their chaperone functions. While the main chaperone-related functions typically are associated with the N-, and P-domain of CRTs [1], a recent study showed that the C-terminal part of AtCRT3 is essential for retention of the defective BRI1-9 [29]. This raises the interesting point that chaperone, or at least chaperone-related retention, mechanisms may also be present in the C-domain of the CRTs.

Thus, in agreement with several recent reports [27] to [29] our data suggest a significantly diverged role of AtCRT3 from that of AtCRT1a and CRT1b, as demonstrated by the observed strict requirement of the former for the biogenesis of EFR. However, our data also suggest a minor role of AtCRT1a and CRT1b for the establishment of full EFR functions (Figure 9; [27]), implying functional interactions between these two types of CRTs in plants. Further experimentation is needed to elucidate the mechanistic basis for diversified yet mutually supporting functions of CRTs in plants.

## Materials and Methods

### Plant material and genetic analysis

Arabidopsis seeds with T-DNA insertion in *AtCRT1a*, *CRT1b* and *CRT3* were obtained from the Arabidopsis Biological Resource Center (ABRC; http://arabidopsis.org; [43]). The lines used were SALK_055452 (*Atcrt1a*) [11], SALK_062083 (*Atcrt1b-1*), SAIL_ 662_DO5 (*Atcrt1b-2*), SALK_051336 (*Atcrt3*). PCR was performed, with primer sequences generated against the genomic regions flanking the insert and a standard primer for the 3′ end of the insertion sequence, to obtain homozygous insertion lines (Table S1). Reciprocal crosses were undertaken to generate the *Atcrt1a crt1b* double mutant (SALK_055452 with SALK_062083). RT-PCR and western blotting, as described below, was used to ensure that the transcript in the insertion lines was not present (Table S1).

Hypocotyl length was first crudely measured using a 0.5 mm marks, and subsequently confirmed using the software Leica Application Suite (Version 2.8.1, Leica Microsystems CMS, GmBH, Switzerland) with seedlings photographed using a Leica stereomicroscope (Leica MZ 12.5) equipped with a Leica DFC420 digital camera.

### Computational analysis of CRT protein sequences

Amino acid sequences of AtCRT1a, CRT1b and CRT3 and human CRT1 and CRT2 (GenBank accession no. AAC49695, AAK74014, AAC49697, AAA51916, NP_659483, respectively) were obtained from National Center for Biotechnology Information (NCBI; www.ncbi.nlm.nih.gov). Alignment was done using ClustalW, MacVector 8.0 software (Oxford Molecular group plc, UK).

Microarray datasets were obtained from NASCarrays (http://affymetrix.arabidopsis.info/narrays/experimentbrowse.pl), and were downloaded and plotted in excel. Signals with values below 100 were excluded from the analysis.

Co-expression networks have been taken, and modified, from AraGenNet ([44]; http://aranet.mpimp-golm.mpg.de/aranet).

### Isolation of RNA and RT-PCR

RNA was isolated from Arabidopsis tissues with Qiagen RNeasy Plant Mini Kit (Qiagen Inc., Valencia, CA, USA). One µg RNA was DNase digested (Promega, Madision, WI, USA) followed by cDNA synthesis with iScript cDNA Synthesis Kit (BIO-RAD, Hercules, Ca, USA). RT-PCR primers (Table S1) were designed over exon-exon junctions and set on a T_m_ ranging from 58–62°C with a GC-content to 45–55%. Actin was used as a control to assure intact RNA.

### GUS-construct and staining

The tissue-level expression pattern of the *AtCRT1a, CRT1b* and *CRT3* genes were studied using the β -glucuronidase (GUS) reporter gene. A genomic DNA fragment approximately extending 1.5-kb upstream from the ATG starting codon for the genes were amplified and inserted, using BamHI and NcoI, in front of the GUS gene in the pCAMBIA1305 GUS-Plus vector (Table S1). The constructs were transformed into wild-type plants by *Agrobacterium*-mediated transformation [45]. Transgenic plants were selected on hygromycin, and used for GUS reporter analyses. Tissues were incubated in GUS staining solution (100 mM sodium phosphate, pH 7.0, 10 mM EDTA, 1 mM ferricyanide, 1 mM ferrocyanide, and 1 mM 5-bromo-4-chloro-3-indolyl β-D-glucuronic acid) at 37°C. After clearing in 70% ethanol, the tissues were observed for GUS staining under a dissecting microscope.

### Induction of expression as assessed by promoter-GUS lines

Arabidopsis seeds were germinated and grown for three days on ½ Murashige and Skoog (MS) medium with 0.8% (w/v) agar and 1% (w/v) sucrose. Seedlings were subsequently transferred to slides coated with ½ Murashige and Skoog (MS) medium with 0.8% (w/v) agar and 1% (w/v) sucrose, and grown for two additional days. The slides were immersed vertically in liquid covering the lower parts of the roots for 12 and 24 h. The liquids contained either 150 mM glucose, 150 mM sucrose, 150 mM mannitol, 10 µg/ml tunicamycin, 10 mM DTT, or 250 mM salicylic acid, respectively. The seedlings were then immersed in GUS staining solution and treated as described above.

### Antibodies

Peptide anti-rabbit antibodies were raised against the AtCRT1a (GDDSDNESKSEETKEAE), the CRT1b (SEETSEKDATAHD), and for the CRT3 as described in [28]. An N-terminal cysteine was added to allow affinity purification on SulfoLink Coupling Gel (Pierce Biotechnology) according to manufacturer's protocol. The buffer was exchanged to PBS (137 mM NaCl, 2.7 mM KCl, 10vmM Na_2_HPO_4_, 2 mM KH_2_PO_4_, pH 7.4) using a PD10 column (Pharmacia Amersham). The pre-immune sera for the respective antibodies were handled the same way. The specificity of the peptide antibodies were confirmed using mutants for the respective CRTs. The CRT maize antibody were used in dilution 1∶10 000 [24].

### Immunofluorescence

Two-week-old roots from seedling grown in a liquid culture were fixed for 3 h in 4% (w/v) paraformaldehyd, 0.05% (w/v) glutaraldehyd in 0.1 M potassium phosphate buffer, pH 5.7. The sections was washed several times in 0.1 M potassium phosphate buffer, and then put in a 25% (w/v) sucrose in potassium phosphate buffer in 4°C over night. The roots were cryo-sectioned to 14 µm sections and washed in PBS with 0.25% (w/v) Triton X-100, 2×15 min. They were incubated with the respective antibody and pre-immune serum, diluted 1∶500 in PBS with 0.25% (w/v) Triton X-100 and 1% (w/v) BSA, over night in a moist chamber. The sections were washed in PBS, 2×15 min and than incubated with Alexa Fluor 488 antibody (Molecular Probes), 1∶200 in PBS with 1% BSA for 1 h in darkness in a moist chamber. The sections were washed as before in PBS and then mounted with glycerol and PBS at a ratio of 9∶1. Microscopy was performed in a wide field fluorescent microscopy with GFP filter.

### Immunohistochemistry using transmisson electron microscopy

Roots for TEM were fixed for 24 h at 4°C in 4% (w/v) paraformaldehyde, 0.25% (w/v) glutaraldehyde in 0.1 M potassium phosphate buffer, at pH 5.7. The roots were washed several times in 0.1 M potassium phosphate buffer and embedded in lowicryl according to [46]. Fifty µm thick sections on nickel grids were blocked for 1 h in PBS with 1% BSA and 0.1% Tween 20. The grids were then added to primary antibodies, (in PBS with 0.1% BSA and 0.01% Tween 20, diluted 1∶50 AtCRT1b and 1∶200 callose [1-3-β-glucan (Biosupplies, Australia)] at 4°C 16 h. The grids were washed three times in PBS and incubated with a secondary gold antibody (Aunoprobe EM GAR15, or AunoProbe EM GAM G10) ratio 1∶30 diluted in the same buffer as the primary antibody for 3 h in room temperature and then washed with PBS, fixed with 1% (w/v) glutaraldehyde for 5 minutes and washed with dH_2_O three times for one h. The grids were stained with 2% uranyl acetate for 30 min, and with lead citrate according to [47] for 3 minutes. TEM was performed in a JEOL 1230 transmission electron microscopy.

### Visualization of the CFP-tagged AtCRT3

An *AtCRT3* cDNA was introduced into a TOPO cloning vector (Invitrogen, CA, USA) using primers according to Table S1. An endogenous restriction site close to the C-terminal end (AgeI) was used to clone in the CFP (Table S1), resulting in the plasmid pTOPO:AtCRT3-CFP. The AtCRT3 tagged CFP was cut out using BstEII and SpeI and was introduced into a pCAMBIA1302 after the 35S promoter. The resulting plasmid was introduced into tobacco leaves via agrobacterium leaf infiltration (culture OD_600_ = 19) essentially according to [48]. Confocal microscopy was carried out on a Leica SP5 TCS confocal fitted to a Leica BM6000B upright microscope equipped with a 63X water objective. CFP was visualized using a 405 nm Lazer controlled by Leica Application Suite Advanced Fluorescence imaging software. For the CRT3-CFP images, one µm Z-stack sections were recorded (36 sections in total), which were merged to a single Z-stack image using the Leica Application Suite Advanced Fluorescence imaging software.

### Generation of transgenic *AtCRT3* mouse embryonic fibroblasts

A full-length *AtCRT3* cDNA (GenBank Accession U66345) or mutated *AtCRT3* were cloned into a pcDNA3.1/Zeo vector containing a hemagglutinin epitope (HA) tag in the C terminus, upstream of the ER retention signal (HDEL) of *AtCRT3* to generate pcDNA-*AtCRT3*-HA. Site-specific mutagenesis was carried out for the mutated *AtCRT3* using a QuickChange II Site-directed mutagenesis kit (Stratagene, La Jolla, Ca, USA). Two amino acids were mutated from Asn^268^ to Asp^268^ and Leu^270^ to Glu^270^. The mutated amino acids were placed at N^268^ and L^270^ respectively. Throughout this work, the mutated *AtCRT3* will be referred to *AtCRT3m*. Wild-type and *crt^−/−^* mouse embryonic fibroblasts were grown at 37°C in a 5% CO_2_ environment in Dulbecco's modified Eagle's medium containing 10% (w/v) fetal bovine serum and 1% (w/v) penicillin streptomycin-glutamine [3]. *crt^−/−^* cells were transfected with pcDNA *AtCRT3*-HA or pcDNA *CRT3m*-HA using Effectene Transfection Reagent (Qiagen Inc., Valencia, CA, USA), and stable transfected cell lines were selected in the presence of 400 µg ml*^−^* ^1^ zeocin. Five of these cell lines were analyzed more in detail with respect to AtCRT3 or AtCRT3m protein expression, subcellular localization, and effects on Ca^2+^ homeostasis and putative protein folding.

### SDS-PAGE and immuno-blotting

SDS-PAGE was carried out essentially according to [49] with a Bio-Rad Mini-Protean II electrophoresis system. For mouse embryonic fibroblasts, the cells were lysed with RIPA buffer (50 mM Tris-HCl, pH 7.5, 150 mM NaCl, 1 mM EGTA, 1 mM EDTA, 1% (w/v) Triton-X100, 0.5% (w/v) deoxycholic acid, 0.1% (w/v) SDS, 1 mM benzamidine, 1 mM PMSF, 0.025 mg ml^−1^ aprotinin, 0.01 mg ml^−1^ pepstatin, 0.05 mg ml^−1^ E-64, 0.025 mg ml^−1^ leupeptin and 0.1 mg ml^−1^ tosyl phenylalanyl chloromethyl ketone (TPCK)) and proteins were solubilized by addition of sample buffer (125 mM Tris HCl, pH 6.8, 4% (w/v) SDS, 20% (v/v) glycerol, 5% β-mercaptoethanol and 0.02% bromphenol blue), and separated using a 12% SDS-polyacrylamide gel. For immunoblotting analysis, proteins were electrophoretically transferred to a PVDF membrane (Millipore) for 1 h at 100 V. After transfer, the membrane was blocked with 5% blocking solution (5% (w/v) skimmed milk powder in 10 mM Tris-HCl, pH 8.0, 150 mM NaCl), washed in 10 mM Tris-HCl, pH 8.0, 150 mM NaCl and 0.05% (w/v) Tween-20. For mouse embryonic fibroblasts the blotting membranes were probed with antibodies against the HA-tag (1∶1000). The HA-tag antibodies were detected with anti-rat IgG, horseradish peroxidase secondary antibodies (1∶5000). For plants ten or twenty (indicated in figure legends) µg protein from microsomal fractions of cell suspension or homogenate from leaves was used and incubated with the respective antibody [maize CRT (1∶10,000), or peptide antibodies for the respective CRT (1∶1,000)], and a secondary antibody [1∶10,000 (antirabbit IgG from Amersham Biosciences)] linked with horseradish peroxidase. Detection was obtained using the Enhanced Chemiluminescence protocol (BioRad Laboratories) and developed on hyperfilm ECL (Amersham Biosciences). The anti-EFR, and anti-FLS2 sera used was described in [28].

### Immunohistochemistry of mouse embryonic fibroblasts expressing *AtCRT3*

To localize AtCRT3 and CRT3m intracellularly in mouse embryonic fibroblasts, cells were cultured on coverslips, washed with PBS (5 mM potassium phosphate buffer, pH 7.5, 150 mM NaCl), fixed with 4% (w/v) formaldehyde, extracted with 0.1% (w/v) saponin, 2% (w/v) milk powder in PBS, and incubated with HA-tag monoclonal antibodies (1∶50) and PDI polyclonal antibodies (1∶500). The HA-tag antibodies were detected with fluorescence Alexa Fluor anti-rat secondary antibodies at 546 nm and the PDI antibodies with fluorescence Alexa Fluor anti-rabbit secondary antibodies at 488 nm. The nucleus was stained with DAPI (4,6-diamidino-2-phenylindole), and visualized at 350 nm. All imaging was done on a Zeiss confocal microscope.

### Analysis of N-linked glycans in mouse embryonic fibroblasts

Mouse embryonic fibroblasts were lysed with RIPA buffer and used for glycosidase analysis, SDS-PAGE and immunoblotting. Recombinant PNGase F was used for the N-linked glycosidase treatment (New England Biolabs, Beverly, MA). De-glycosylation was carried out essentially according to the manufacturer's protocol under native conditions

### Measurements of ER Ca^2+^ capacity

Mouse embryonic fibroblasts were cultured for 50 h in the presence of 55 µCi ^45^Ca^2+^ [3]. Cells were then washed twice with 5 mM EDTA in PBS, and then once with 5 mM EDTA in culture medium (without fetal bovine serum), detached from Petri dishes by trypsinization (0.25% (w/v) trypsin and 0.02% (w/v) EDTA in Ca^2+^/Mg^2+^-free PBS), and finally resuspended in Ca^2+^-free buffer (143 mM NaCl, 6 mM KCl, 1 mM MgSO_4_, 20 mM Hepes-NaOH, pH 7.4, 0.1% (w/v) glucose and 0.1 mg mL*^−^* ^1^ sulfinpyrazone). For ^45^Ca^2+^ release experiments, aliquots of 5×10^6^ cells mL*^−^* ^1^ were preincubated for 3 min at 37°C followed by treatment with thapsigargin or ionomycin for 4 min at 37°C. Cells were then pelleted by centrifugation (9,600*g* for 3 min) and the radioactivity in the supernatant was measured in a Beckman LS 7800 scintillation counter. Background values, i.e. radioactivity recovered in the supernatant in control incubations without additions of thapsigargin or ionomycin, were subtracted prior to presentations in Figures.

### Fluorescence Ca^2+^ measurements of mouse embryonic fibroblasts

Cells were incubated with 2 µmol L*^−^* ^1^ Fura 2-AM (Sigma-Aldrich) in Ca^2+^ buffer (143 mM NaCl, 6 mM KCl, 1 mM MgSO_4_, 20 mM Hepes-NaOH, pH 7.4, 0.1% (w/v) glucose, 1 mM CaCl_2_ and 0.1 mg ml*^−^* ^1^ sulfinpyrazone) essentially as described in [3], washed with PBS, trypsinized (0.25% (w/v) trypsin and 0.02% (w/v) EDTA in Ca^2+^/Mg^2+^-free PBS), centrifuged, washed in Ca^2+^-free buffer (143 mM NaCl, 6 mM KCl, 1 mM MgSO_4_, 20 mM Hepes, pH 7.4, 0.1% (w/v) glucose and 0.1 mg ml*^−^* ^1^ sulfinpyrazone), centrifuged, and resuspended in Ca^2+^-free buffer at 1×10^6^ cells ml*^−^* ^1^. The cells were transferred to a cuvette and 2 mM EGTA was added. Fluorescence was measured at λ_ex_ = 340 nm or 380 nm and λ_em_ = 510 nm. When the resting free cytoplasmic Ca^2+^ level of the cells (basal Ca^2+^ level) was reached the cells were stimulated with 600 nM bradykinin (Figure 5H). Thapsigargin (300 nM), a SERCA (ER Ca^2+^-ATPase) inhibitor, was later added to the cells to measure the Ca^2+^ store in the ER and to control that no Ca^2+^-release of BK-induced cells were due to incomplete Ca^2+^ storage functions in the ER (Figure 5H). To measure the store-operated Ca^2+^ influx, 2 mM CaCl_2_ was added to the cells. Ionomycin (7.5 µM) together with CaCl_2_ (4 mM) was added to the cells to obtain the max value and to obtain the minimum value 32 mM EGTA, 24 mM Tris-HCl, pH 7.4 and 0.4% (w/v) Triton X-100 were added to the cells.

### Assessment of cell adhesion of the mouse fibroblasts

Mouse fibroblasts of wild-type, *crt^−/−^* and CRT-deficient mouse fibroblasts expressing *AtCRT3* or *CRT3m* were plated, with a density of 300 000 cells per line, in 2.5 mm diameter tissue culture dishes and grown at 37°C in a 5% CO_2_ environment in Dulbecco's modified Eagle's medium containing 10% (w/v) fetal bovine serum and 1% (w/v) penicillin streptomycin-glutamine for 16 h before phase contrast image and cell counting.

### Tunicamycin treatments of Arabidopsis seedlings

Arabidopsis seeds were germinated and grown on ½ Murashige and Skoog (MS) medium with 0.8% (w/v) agar at 23°C with 16 h of light (100 µE m*^−^* ^2^ s*^−^* ^1^) and were transferred after one week to ½ MS medium with 0.8% agar containing 0.1 µg mL*^−^* ^1^ tunicamycin. After 8 days of tunicamycin treatment the plants were photographed and fresh weight was measured. Dark grown hypocotyls were grown for 6 days on H_2_O with 0.8% agar in 23°C in dark.

### Real-time PCR

Real-time PCR analysis (Table S1) was performed on six-day-old light grown seedlings grown on 1MS supplemented with 1% sucrose. The real-time PCR was executed using a Step-One plus system (Applied Biosystems, CA, USA), following the outline presented in [50]. The relative numbers for CT were normalized to the house keeping gene ubiquitin10 (UBQ10) that was used as control. For visualization purposes we calculated the 40 cycles minus ΔCT, i.e. the value obtained from the normalized CT value, and therefore obtained positive values for the ΔCT.

### Complementation of *Atcrt1b* and *Atcrt1a crt1b* double mutants

A full-length *AtCRT1a* cDNA (GenBank Accession U66343) or *AtCRT3* cDNA (GenBank Accession U66345) was cloned into a p35SBARN binary vector ([51]; Table S1). The construct was transformed into electro-competent *Agrobacterium tumefaciens* GV3101, and transformed into *Atcrt1b* mutant by floral dip [45]. Transformed plants were grown on ½ MS medium with 0.8% agar containing 50 µg ml*^−^* ^1^ kanamycin (resistance conferred by *Atcrt1a* and *crt1b* T-DNA insertion mutation) and 25 µg ml*^−^* ^1^ BASTA (resistance conferred from *AtCRT1a* or *CRT3* transgene) for selection to homozygous seed lines. Expression of the transgene was monitored by RT-PCR analyses or western blot.

### PAMP assays

Anthocyanin content, and ROS accumulation were analyzed as described in [28], using six-day-old seedlings (anthocyanin assays) or leaf discs (5 mm in diameter) for ROS accumulation).

### Statistics

Statistical significance of differences between treatments or measurements of different cell lines was assessed by Student's *t-test* (Microsoft Office Excel, Microsoft Corporation, Redmond, WA). The number of replicates and the level of significance is given in text or table.

## Supporting Information

Figure S1 Expression analyses of AtCRT1a, CRT1b, and CRT3. A. Microarray signal values for AtCRT1a plotted against values for AtCRT1b over 2332 microarray datasets obtained from the Nottingham Arabidopsis Science Center (NASC; http://arabidopsis.info/). B. Expression analysis of AtCRT1a and CRT3 in different Arabidopsis tissues using a semi-quantitative RT-PCR. Upper panel AtCRT1a, middle panel AtCRT3, and lower panel ACTIN that was used as control.
(5.69 MB EPS)

Figure S2 Expression, glycosylation status and localization of a mutated AtCRT3 in *crt^−/−^* mouse fibroblasts. A. Analysis of glycosylation status of AtCRT3 and CRT1a transfected into *crt^−/−^* mouse fibroblasts, before and after treatment with N-glycosidase F (PNGase F) for 1 h under native conditions, and probed with HA-tag antibodies. -, without PNGase F and +, with PNGase F. B. Western blot analysis of AtCRT3m produced in *crt^−/−^* mouse fibroblasts. The PVDF membranes were probed with anti-HA-tag antibodies. *crt^−/−^*, CRT-deficient mouse fibroblasts; Empty vector, mock transfected *crt^−/−^* mouse fibroblasts; other cell lines represent AtCRT3m transfected into *crt^−/−^* mouse fibroblasts. C to F. Immunolabeling of AtCRT3m-24 produced in CRT-deficient mouse cells with anti-HA-tag; (C) anti-HA for AtCRT3m, (D) anti-PDI (ER-marker), and (E) DAPI (nucleus-marker). F. Overlay, the yellow color indicates identical localization of ER-marker and AtCRT3.
(3.37 MB EPS)

Figure S3 Cellular Ca^2+^ content in AtCRT3 expressing fibroblast lines. A. The Ca^2+^ content was determined by incubation with 45Ca^2+^ followed by addition of ionomycin to determine total cellular Ca^2+^ levels. The background values (counts min-1 in supernatant before addition of thapsigargin or ionomycin) were subtracted. Results represent the average ± SE of three independently performed experiments. B and C. Protein loading controls for Fig. 8B (B), and Fig. 9F (C).
(2.14 MB EPS)

Figure S4 Real-time PCR analysis of different genes for putative ER chaperones in the Atcrt mutants. The relative expression values of the different genes are displayed as 40 minus the normalized deltaCT value. * denote the N/A assay for AtCRT1a in the Atcrt1a and Atcrt1a crt1b mutants.
(0.98 MB EPS)

Table S1 (0.07 MB DOC)
