# Supplementary material for: Higher Plant Calreticulins Have Acquired Specialized Functions in Arabidopsis
Source: PLoS One. 2010 Jun 28;5(6):e11342. doi: 10.1371/journal.pone.0011342 (PMC2893204; doi:10.1371/journal.pone.0011342)
Supplement: Table S1 — (0.07 MB DOC) [file pone.0011342.s005.doc]

Table S1. Primers used for different genomic and expression analyses.

| **Locus/construct** | **Purpose** | **Direction** | **Cut** | **Primer sequence (5’ to 3’)** |
| --- | --- | --- | --- | --- |
| At1g09210 (AtCRT1b) | (RT-PCR) | For |  | ACCTGCGGAATCTGATGCTG |
| At1g09210 (AtCRT1b) | (RT-PCR) | Rev |  | ATCATGAGCGGTGGCGTCT |
| At1g56340 (AtCRT1a) | (RT-PCR) | For |  | AGCACAAGGATGCGGAGA |
| At1g56340 (AtCRT1a) | (RT-PCR) | Rev |  | TCAGAGTCGGATTCAGC |
| At1g08450 (AtCRT3) | (RT-PCR) | For |  | GTGGATGACTATTTTGCCC |
| At1g08450 (AtCRT3) | (RT-PCR) | Rev |  | CACCTTCTTCCCGTGCTAT |
| ACTIN | (RT-PCR) | For |  | tcttaacccaaaggccaaca |
| ACTIN | (RT-PCR) | Rev |  | cagaatccagcacaataccg |
| At1g09210 (AtCRT1b) | SALK_062083 | For |  | AGACGCCAATAAACCTTTTGG |
| At1g09210 (AtCRT1b) | SALK_062083 | Rev |  | TTTGGTCCTGATATCTGTGGC |
| At1g08450 (AtCRT3) | SALK_051336 | For |  | AGGAGCATTTCGAAGGTGAAG |
| At1g08450 (AtCRT3) | SALK_051336 | Rev |  | CAAGAGTCCGGTTTTTGTTGC |
| SALK insert primer |  |  |  | CGCTTTCTTCCCTTCCTTTCTC |
| SAIL insert primer |  |  |  | TGACGCCATTTCGCCTTTTC |
| AtCRT1a complement |  | For |  | agcacaaggatgcggaga |
| AtCRT1a complement |  | Rev |  | tcagcgtcggattcagc |
| AtCRT3 complement |  | For |  | gtggatgactattttgccc |
| AtCRT3 complement |  | Rev |  | gaccttcttcccgtgctat |
| CRT1a promoter |  | For | BamHI | TATAGGATCCGAAGACGAATACCTAATGGCTGTG |
| CRT1a promoter |  | Rev | NcoI | ATTGCCATGGACTCAGATCGGAGCTTCACACG |
| CRT1b promoter |  | For | BamHI | CAGAGGATCCGGAAACAGATGGATGTTAGAGG |
| CRT1b promoter |  | Rev | NcoI | ATTGCCATGGAAAACTCAGATCGGAGCTTCCC |
| CRT3 promoter |  | For | BamHI | CAATGGATCCATGGCAATCAAGAATATGTTGC |
| CRT3 promoter |  | Rev | NcoI | TTTTCCATGGTTTCTCACTTCATCATGGTTACTG |
| CRT3 for CFP |  | For | SpeI | CCATG**actagt**ATGGGATTACCTCAAAATAAGCTC |
| CRT3 for CFP |  | Rev | BstEII | ccca**ggtcacc**TCAtagctcgtcatggtaatc |
| CFP |  | For | AgeI | tcgccaAc**CGg**Ttgagcaagggcgaggagc |
| CFP |  | Rev | AgeI | gccgc**accggt**tgtacagctcgtccatgc |
| At1g56340 (AtCRT1a) | Real-time PCR | For |  | GACAATGTCTTGGTCAGCGATG |
| At1g56340 (AtCRT1a) | Real-time PCR | Rev |  | GCTTCGTCAAATGCTGCTTTCT |
| At5g61790 (AtCNX1) | Real-time PCR | For |  | CCTCAAGCTTATCTTTGGTGGC |
| At5g61790 (AtCNX1) | Real-time PCR | Rev |  | TTCCTTCTTCTCCGCCTCATC |
| At4g24190 (AtSHD) | Real-time PCR | For |  | AGTAGCCGACGAGGAAATCGA |
| At4g24190 (AtSHD) | Real-time PCR | Rev |  | CGGCTTCAATGTTTAGACCACC |
| At1g21750 (AtERp57a) | Real-time PCR | For |  | TCAAATCAGCGAGCGGAAAC |
| At1g21750 (AtERp57a) | Real-time PCR | Rev |  | AGGCTCTCCAACTGTGTCCTTG |
| At5g28540 (AtBiP1) | Real-time PCR | For |  | AAGTTTCCGATATGGCTCGC |
| At5g28540 (AtBiP1) | Real-time PCR | Rev |  | AGCCTCTTCTTTTGCAGTGGA |
